# Modification in mitochondrial function is associated with the *FADS1* variant and its interaction with alpha-linolenic acid-enriched diet—An exploratory study

**DOI:** 10.1016/j.jlr.2024.100638

**Published:** 2024-08-31

**Authors:** Maija Vaittinen, Mariana Ilha, Ratika Sehgal, Maria A. Lankinen, Jyrki Ågren, Pirjo Käkelä, Kirsi A. Virtanen, Markku Laakso, Ursula Schwab, Jussi Pihlajamäki

**Affiliations:** 1 Institute of Public Health and Clinical Nutrition, University of Eastern Finland, Kuopio, Finland; 2 Department of Neurosurgery, University of Pennsylvania, Philadelphia, PA; 3 Department of Experimental Diabetology, German Institute of Human Nutrition (DIfE), Potsdam, Germany; 4 Institute of Biomedicine, School of Medicine, University of Eastern Finland, Kuopio, Finland; 5 Department of Surgery, University of Eastern Finland and Kuopio University Hospital, Kuopio, Finland; 6 Turku PET Centre, Turku University Hospital, Turku, Finland; 7 Institute of Clinical Medicine, Internal Medicine, University of Eastern Finland, Kuopio, Finland; 8 Department of Medicine, Kuopio University Hospital, Kuopio, Finland; 9 Department of Medicine, Endocrinology, and Clinical Nutrition Kuopio University Hospital, Kuopio, Finland

**Keywords:** adipocytes, alpha-linolenic acid, dietary fat, FADS1, fatty acid oxidation, human adipose-derived stromal cell, lipids/oxidation, mitochondria, omega-3 fatty acids, polyunsaturated fatty acid

## Abstract

Fatty acid desaturase (*FADS1*) variant-rs174550 strongly regulates polyunsaturated fatty acid (PUFA) biosynthesis. Additionally, the *FADS1* is related to mitochondrial function. Thus, we investigated whether changes in mitochondrial function are associated with the genetic variation in *FADS1* (rs174550) in human adipocytes isolated from individuals consuming diets enriched with either dietary alpha-linolenic (ALA) or linoleic acid (LA). Two cohorts of men homozygous for the genotype of *FADS1* (rs174550) were studied: FADSDIET2 dietary intervention study with ALA- and LA-enriched diets and Kuopio Obesity Surgery study (KOBS), respectively. We could demonstrate that differentiated human adipose-derived stromal cells from subjects with the TT genotype had higher mitochondrial metabolism compared with subjects with the CC genotype of *FADS1*-rs174550 in the FADSDIET2. Responses to PUFA-enriched diets differed between the genotypes of *FADS1*-rs174550, showing that ALA, but not LA, -enriched diet stimulated mitochondrial metabolism more in subjects with the CC genotype when compared with subjects with the TT genotype. ALA, but not LA, proportion in plasma phospholipid fraction correlated positively with adipose tissue mitochondrial-DNA amount in subjects with the CC genotype of *FADS1*-rs174550 in the KOBS. These findings demonstrate that the *FADS1*-rs174550 is associated with modification in mitochondrial function in human adipocytes. Additionally, subjects with the CC genotype, when compared with the TT genotype, benefit more from the ALA-enriched diet, leading to enhanced energy metabolism in human adipocytes. Altogether, the *FADS1*-rs174550 could be a genetic marker to identify subjects who are most suitable to receive dietary PUFA supplementation, establishing also a personalized therapeutic strategy to improve mitochondrial function in metabolic diseases.

Obesity is a serious public health problem worldwide (1) characterized by insulin resistance (IR), dyslipidemias, and mitochondrial dysfunction, among others (2, 3). Of these risk factors, mitochondrial dysfunction is of particular interest since compelling evidence suggests that stimulating mitochondrial activity could be used as a therapeutic strategy to tackle obesity and improve insulin sensitivity (4). However, many of the proposed strategies to stimulate mitochondrial function in adipose tissue are confirmed in vivo or in vitro, and approaches to stimulate mitochondrial function in white adipose tissue are scarce in humans (5).

Lifestyle modifications aiming at changing the quality of dietary fatty acids (FAs) have been suggested to have important roles in the development of obesity, IR, type 2 diabetes (6, 7), and mitochondrial function (7, 8). Specifically, omega (n)-3 polyunsaturated fatty acids (PUFAs) and their lipid mediators, synthesized from the precursor alpha-linolenic acid (ALA), including eicosapentaenoic acid (EPA) and docosahexaenoic acid (DHA), have been shown to improve insulin sensitivity, stimulate mitochondrial function, and decrease diet-induced obesity (7, 8). On the other hand, a Western diet with a high n-6/n-3 PUFA ratio dictates the availability of n-6 PUFAs, like linoleic acid (LA) and its derivative arachidonic acid (AA) (9). LA has been shown to have beneficial effects on human health; however, recent research has questioned this by demonstrating harmful effects on mitochondrial function and lipid metabolism (10, 11, 12). Therefore, n-3 PUFAs rather than n-6 PUFAs could induce more beneficial changes in mitochondrial function.

The synthesis of n-3 and n-6 PUFAs is mediated by the enzymes delta-5 (D5D) and delta-6 desaturases (D6D), encoded by the fatty acid desaturase (*FADS) 1* and *FADS2* genes, respectively (13). D5D enzyme is known to function in the endoplasmic reticulum to catalyze the biosynthesis of PUFAs and also participate in cytoplasmic nicotinamide adenine dinucleotide recycling (14). However, recent evidence suggests that D5D is also localized in mitochondria (15) yet unspecified function. Although *FADS1* has been shown to be associated with reactive oxygen species generation and induction of mitochondrial-mediated apoptosis (16), features involved in mitochondrial function.

Importantly, recent studies have demonstrated that genetic variations in the *FADS* genes are associated with glucose homeostasis, PUFA composition, the development of metabolic dysfunction-associated steatotic liver disease (13, 17, 18), and the risk of type 2 diabetes (19). Specifically, the *FADS1* variant (rs174550), which is one of the risk variants for hyperglycemia and type 2 diabetes, has been shown to strongly associate with PUFA composition and PUFA-derived lipid mediator metabolism (20), proposing that the *FADS1*-rs174550 variant could regulate PUFA-induced changes in disease susceptibility. Furthermore, our previous results demonstrated that the *FADS1*-rs174550 could modify LA-induced changes in plasma high-sensitive C reactive protein (hs-CRP) concentration (21, 22), and adipose tissue inflammation (23), suggesting that this genetic variation in the *FADS1* gene may directly modify PUFA-induced changes in inflammation. However, the role of *FADS1*-rs174550 in mitochondrial function in human adipocytes is unknown. Thus, we aim to clarify whether changes in mitochondrial function are associated with the genetic variation in *FADS1* (rs174550) in human adipose-derived stromal cells (hADSCs) isolated from abdominal subcutaneous adipose tissue (scAT) of individuals homozygous (CC: minor allele vs. TT: major allele) for the *FADS1*-rs174550 variant before and after clinical dietary intervention, the FADSDIET2 study.

## Materials and methods

### Subjects

#### FADSDIET 2

The study design of the main FADSDIET2 intervention (NCT03572205) has been previously described (21). Briefly, 335 healthy, normal-weight men without type 2 diabetes homozygous for the *FADS1* variant (rs174550) were recruited from the METabolic Syndrome In Men (METSIM) study and genotyped using the TaqMan SNP Genotyping Assay (Applied Biosystems). The C allele corresponds to minor with lower desaturase activity, and the T allele corresponds to major with higher desaturase activity. Subjects using anticoagulant treatment and having severe chronic diseases were excluded from the study. Altogether, 130 men participated in the intervention, and 118 of them completed the intervention. During the 8-weeks (wk) intervention period, participants were randomly assigned to consume their habitual diet enriched in LA from sunflower oil (62% LA) or ALA from camelina sativa oil (30%–35% ALA, 16% LA) adjusted to their body mass index. ScAT biopsies and blood samples were taken after overnight fasting at the end of the run-in period (BL: baseline, 0 weeks) and at the end of the LA- or ALA-enriched dietary intervention (8 weeks). This study utilized clinical data and scAT samples from which hADSCs were available (LA-enriched diet: 0 weeks and 8 weeks, TT = 4, CC = 4; ALA-enriched diet: 0 weeks and 8 weeks, TT = 4, CC = 4).

#### KOBS

The present analysis also includes a validation cohort with cross-sectional baseline data from a sub-cohort of 22 male individuals with obesity participating in the Kuopio Obesity Surgery (KOBS) study (24). The subjects were genotyped for the *FADS1*-rs174550 variant using the TaqMan SNP Genotyping Assay [genome-wide association studies-based minor allele (C) frequency 0.2979 (25)] and only the CC (n = 8) and TT genotypes (n = 14) having mitochondrial (mt) DNA data from scAT and plasma FA composition available were included in analyses (clinical characteristics presented in Supplemental Table S1, only males included in the analysis).

The study protocols were approved by the Ethics Committee of the Northern Savo Hospital District (FADSDIET 2: 516/2018, KOBS: 1108/2018) and carried out in accordance with the Helsinki Declaration. Written and oral information was given to the participants, and informed written consent was obtained from all participants.

### Biochemical analyses (lipids, glucose, insulin, and FA profiling), and indirect calorimetry

Total, LDL, and HDL cholesterol and total triglycerides were measured using commercial kits (Thermo Fisher Scientific), and plasma glucose concentration was measured using the Konelab 20XTi Clinical Chemistry Analyzer (Thermo Fisher Scientific) and the enzymatic photometric (glucose hexokinase) method (Thermo Fisher Scientific). Plasma insulin concentration was measured with the chemiluminometric immunoassay method (DiaSorin Liaison Analyzer; DiaSorin GmbH). FA composition in plasma was analyzed according to the previously described method using a gas chromatograph (Agilent Technologies, Inc., Wilmington, DE, USA) equipped with a 25-m free FA phase (FFAP) column (26). Indirect calorimetry (Cosmed Quark resting metabolic rate, RMR, with canopy hood, Cosmed srl, Rome, Italy) was used to determine the energy expenditure of study participants after 12 h of overnight fasting according to the instructions provided by the manufacturer. After 30 min of measurement, the respiratory quotient (RQ) was calculated based on the gas exchange through the breath inside a canopy hood as the ratio of carbon dioxide production (VCO2) to oxygen consumption (VO2). RQ from indirect calorimetry can be used clinically to measure FA oxidation in humans, where high RQ values reflect low FA oxidation (27, 28).

### The isolation of hADSC and cell culture

HADSCs were extracted from fresh scAT samples and were cultured in DMEM/F12 supplemented with 10% fetal bovine serum (FBS) and 1% penicillin/streptomycin (pen/strep) in a humidified incubator at 37°C with 5% CO_2_, as previously described (29). After the second day of post-confluence, the cells were washed with PBS and incubated with adipogenic induction media (AIM: DMEM/F12, 1% pen/strep, 3% FBS, 17 μM pantothenate, 33 μM biotin, 100 nM insulin, 100 nM dexamethasone, 500 μM 3-isobutyl-1-methylxanthine, and 1 μM rosiglitazone) for 7 days to induce hADSC differentiation, modified from (29) with the addition of 3% FBS and without transferrin and triiodothyronine (T3) to prevent white adipocyte browning during the differentiation process, according to Herbers *et al.* (30). After the induction period, the hADSCs were incubated with maintenance media (MM: DMEM/F12, 1% pen/strep, 3% FBS, 17 μM pantothenate, 33 μM biotin, 10 nM insulin, 10 nM dexamethasone) for 14 days. Cells were replenished with either AIM or MM every 3 days. Cells at day 0 (hADSCs) and day 21 (differentiated hADSCs) of differentiation were used for further experiments.

### Mitochondrial respiration

Metabolic flux analyses in hADSCs (day 0) and differentiated hADSCs (day 21) were performed by an XFe96 Extracellular Flux Analyzer (Agilent Technologies) using the Mito Cell Stress Test (Agilent) to explore the effects on different mitochondrial respiration-related parameters: basal, uncoupled, and non-mitochondrial cellular oxygen consumption rates (OCR), proton leak, and the estimate of anaerobic glycolysis (extracellular acidification rate, ECAR), as described previously (31). The respiration data was normalized by the total DNA content of cells (CyQUANT Cell Proliferation Assay, Thermo Scientific).

### mtDNA analysis

The total DNA, including mitochondrial DNA (mtDNA), was extracted using the DNeasy Blood & Tissue Kit (Qiagen), as described earlier (31). mtDNA was quantified with the standard curve method using RT-qPCR (QuantStudio 6 Pro, Applied Biosystems) with primers for three different mtDNA genomic regions (16S rRNA, CYTB, and D-loop) and for three nuclear DNA (ncDNA) regions (APP, HBB, and B2M). The relative mtDNA amount was calculated by taking the ratio between each target mtDNA region and the geometric mean of three nuclear DNA regions (mtDNA/ncDNA). Additionally, the statistical analysis was performed using the geometric mean of three different mtDNA genomic regions relative to nuclear DNA. The primer details for mtDNA and ncDNA are shown in Supplemental Table S2.

### Gene expression

Total RNA was extracted using the RNeasy mini kit (Qiagen), and concentration was measured using the Qubit4 Fluorometer (Thermo Scientific). cDNA was synthesized using the High-Capacity cDNA Reverse Transcription Kit (Applied Biosystems) according to the manufacturer’s protocol. Quantitative RT-PCR was carried out with the QuantStudio6 Pro system (Applied Biosystems) using TaqMan™ Fast Advanced Master Mix (Applied Biosystems) or KAPA SYBR FAST qPCR Universal Master Mix (Kapa Biosystems). Cyclophilin A1 (*PPIA*) and ribosomal protein lateral stalk subunit P0 (*RPLP0*) were used as reference genes for calculating the relative expression of target genes using the formula 2^−ddCt^ in Excel (32). The primer details are shown in Supplemental Table S2.

### Statistical analysis

Results were analyzed using the GraphPad Prism9 software for Windows version 9.2.0 (GraphPad Software, San Diego, CA), and data are given as mean ± standard deviation (SD). The normal distribution of data was tested with the Shapiro–Wilk test and the variables were logarithmically (log) transformed when needed. Grubb’s test was used to examine outliers. Baseline (0 weeks) variables were compared between genotypes using one-way ANOVA, Pearson chi-square test, or independent samples *t* test, when appropriate. Genotype × diet interaction was tested using a repeated-measures general linear model with Bonferroni’s multiple comparison test, and within-genotype comparisons (0 weeks compared with 8 weeks) were tested with a paired *t* test. Correlations between the variables were analyzed using partial correlations adjusted for age and BMI (log-transformed variables). *P* ≤ 0.05 was considered statistically significant.

## Results

### *FADS1*-rs174550 variant modifies PUFA composition and dietary PUFA-induced responses to plasma PUFA composition

The clinical characteristics in the total study population of the FADSDIET2 study have been published previously (22) and for the participants from which hADSCs were available are shown in Table 1. The clinical characteristics did not differ between subjects homozygous for the *FADS1*-rs174550 genotypes at 0 weeks of the study (Table 1). As shown in Table 2, the proportion of AA in plasma PL fraction and the estimated D5D activity indexes, AA:DGLA (di-homo-gamma linolenic acid) and AA:LA, were higher in subjects with the TT genotype compared with the CC genotype at 0 weeks of the study. Next, the effects of the ALA- and LA-enriched diets on PUFA proportions in plasma PL fraction were investigated. The results showed that the ALA-enriched diet increased the proportion of ALA in both genotypes and the proportion of EPA in the TT genotype while the proportion of DHA was reduced in the CC genotype (Supplemental Fig. S1A). The LA-enriched diet increased the proportion of LA in the TT genotype but decreased the proportion of AA in the CC genotype (Supplemental Fig. S1B). Additionally, the proportion of ALA was decreased in the CC genotype, and the proportion of docosapentaenoic acid (DPA) and AA:LA ratio reduced in the LA-enriched diet group in both genotypes of *FADS1*-rs174550 (Supplemental Fig. S1B). Further, there were significant genotype × diet interactions for EPA (*P* = 0.022) in the ALA-enriched diet group and for ALA (*P* = 0.006) in the LA-enriched diet group (Supplemental Fig. S1B). Altogether, these demonstrate that the *FADS1*-rs174550 variant can modify dietary PUFA-induced responses to plasma PUFA composition.

**Table 1:** Main characteristics of the study participants from which hADSCs were available in male subjects homozygous for the genotypes (CC, n = 8; TT, n = 8) of *FADS1*-rs174550 variant at the beginning of the study

	TT (n = 8), Major allele	CC (n = 8), Minor Allele	*P* Value^a^
Age (years)	63.8 ± 4.8	63.5 ± 5.0	0.920
Body Weight (kg)	75.9 ± 8.9	76.3 ± 9.2	0.935
BMI (kg m-2)	24.3 ± 1.4	24.3 ± 1.9	0.967
Waist circumference (cm)	91.6 ± 7.2	92.7 ± 6.4	0.760
fP glucose (mmol L-1)	5.8 ± 0.4	5.6 ± 0.4	0.444
fP insulin (mU L-1)	5.9 ± 2.5	7.2 ± 3.2	0.383
fS total cholesterol (mmol L-1)	5.7 ± 0.9	5.4 ± 0.6	0.475
fS LDL cholesterol (mmol L-1)	3.6 ± 0.8	3.4 ± 0.5	0.460
fS HDL cholesterol (mmol L-1)	1.7 ± 0.4	1.6 ± 0.2	0.933
fS triglycerides (mmol L-1)	1.0 ± 0.5	1.0 ± 0.2	0.937
Use of statins, n (%)	1 (13)	1 (13)	1.0
Use of anti-inflammatory drugs, n (%)	1 (13)	1 (13)	1.0
Use of beta-blockers, n (%)	0 (0)	0 (0)	n/a

Mean ± SD; fP, fasting plasma; fS, fasting serum; FADS1, fatty acid desaturase; hADSCs, human adipose-derived stromal cells.

a One-Way ANOVA for continuous variables or *χ*2 test for categorical variables; n/a, non-available.

**Table 2:** Polyunsaturated fatty acid (PUFA) proportions and estimated enzyme activities in plasma phospholipid (PL) fraction in male subjects homozygous for the genotypes (CC, n = 8; TT, n = 8) of *FADS1*-rs174550 variant at the beginning of the study

PUFA Proportions in plasma PL Fraction (molar Percentage)	TT (n = 8), Major allele	CC (n = 8), Minor Allele	*P* Value^a^
PUFA	41.4 ± 2.3	41.4 ± 0.9	0.995
total n-6	32.7 ± 2.6	31.9 ± 2.0	0.520
Linoleic acid (LA), 18:2n-6	20.4 ± 2.6	22.0 ± 2.6	0.264
Di-homo-gamma linolenic acid (DGLA), 20:3n-6	2.3 ± 0.4	2.3 ± 0.5	0.934
Arachidonic acid (AA), 20:4n-6	9.6 ± 1.0	7.3 ± 1.5	***0.002***
Adrenic acid (AdA), 22:4n-6	0.2 ± 0.1	0.2 ± 0.1	0.346
Osbond acid (ObA), 22:5n-6	0.1 ± 0.1	0.1 ± 0.05	0.802
total n-3	8.8 ± 1.8	9.5 ± 2.0	0.440
Alpha-linolenic acid (ALA), 18:3n-3	0.3 ± 0.2	0.4 ± 0.1	0.363
Eicosapentaenoic acid (EPA), 20:5n-3	2.2 ± 0.7	1.9 ± 0.8	0.392
Docosapentaenoic acid (DPA), 22:5n-3	1.1 ± 0.1	1.0 ± 0.1	0.132
Docosahexaenoic acid (DHA), 22:6n-3	5.1 ± 1.4	6.2 ± 1.3	0.111
Estimated enzyme activities			
AA:DGLA	4.4 ± 1.0	3.3 ± 0.7	***0.030***
AA:LA	0.5 ± 0.1	0.3 ± 0.1	***0.012***
EPA:ALA	8.1 ± 3.5	5.6 ± 3.0	0.145

Bolded with italics, significant.

Mean ± SD; PUFA, polyunsaturated fatty acid; PL, phospholipid; n-, omega; FADS1, fatty acid desaturase.

a One-Way ANOVA for continuous variables.

### *FADS1*-rs174550 variant is associated with modification in mitochondrial respiration and mtDNA amount in differentiated hADSCs

**Figure 1:**
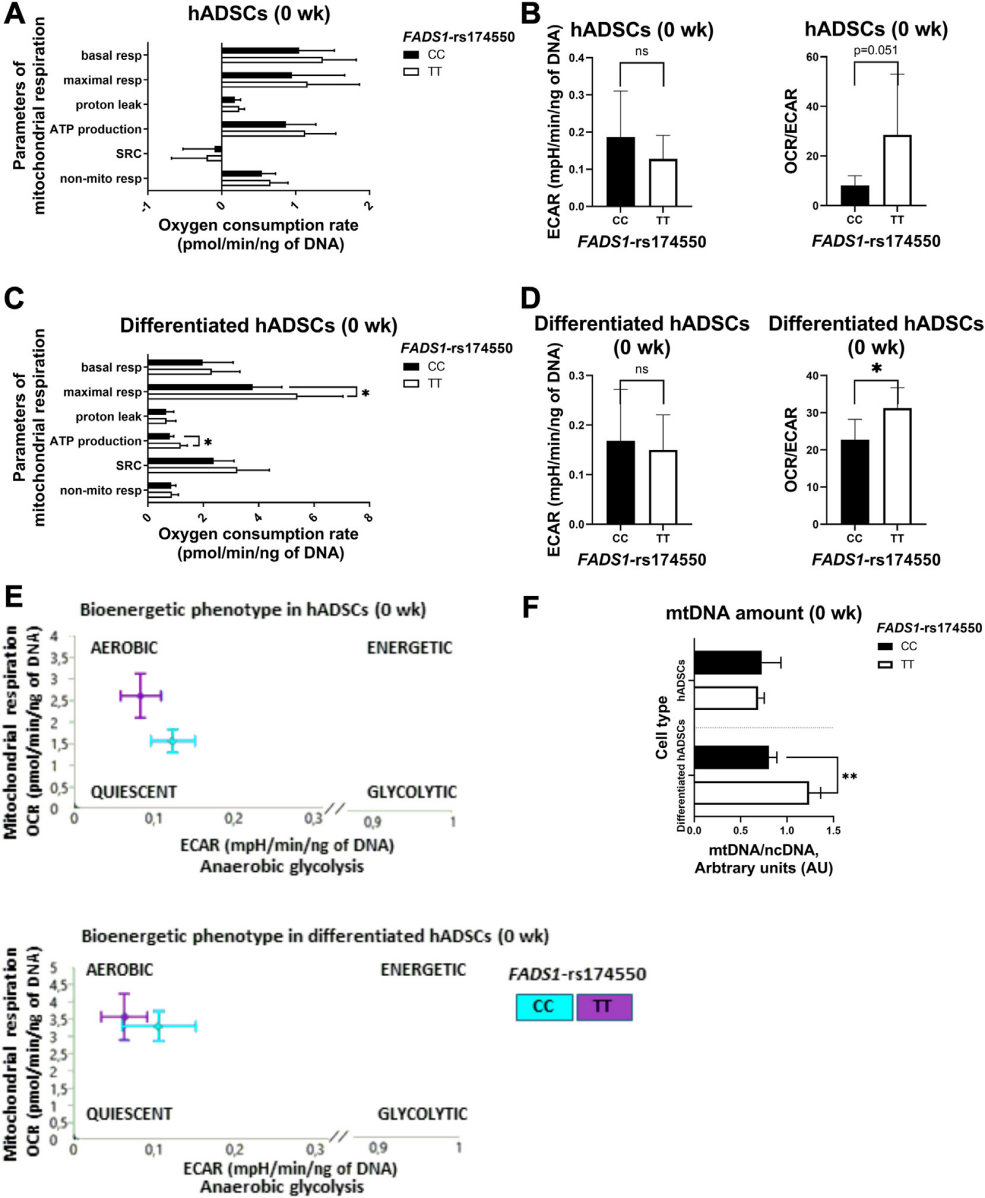
*FADS1*-rs174550 variant associates with modification in mitochondrial respiration and mitochondrial DNA (mtDNA) amount. The effect of *FADS1*-rs174550 variant on mitochondrial respiration (presented as the parameters of mitochondrial respiration: basal respiration, maximal respiration, proton leak, ATP production, spare respiratory capacity (SRC), and non-mitochondrial (non-mito) respiration), ECAR (anaerobic glycolysis), and OCR/ECAR ratio in (A, B) hADSCs and (C, D) differentiated hADSCs at 0 weeks of the FADSDIET2 study. E: Schematic representation of cellular bioenergetics phenotype profile in hADSCs and differentiated hADSCs in subjects homozygous for the *FADS1*-rs174550 variant at 0 weeks of the FADSDIET2 study. F) The effect of *FADS1*-rs174550 variant on the mtDNA amount in hADSCs and differentiated hADSCs in subjects homozygous for the *FADS1*-rs174550 variant at 0 weeks of the FADSDIET2 study. Values are presented as mean ± SD, n = 8 independent biological replicates/genotype from male subjects, including 8 technical replicates/biological replicate. Statistics: independent samples *t* test for differences between genotypes (∗*P* < 0.05, ∗∗*P* < 0.01). FADS1, fatty acid desaturase 1; hADSCs, human adipose-derived stromal cells; OCR, oxygen consumption rate; ECAR, extracellular acidification rates.

As the D5D/FADS1 protein is shown to be localized in mitochondria (15), the ability of the *FADS1*-rs174550 variant to modify mitochondrial bioenergetics in hADSCs and differentiated hADSCs at 0 weeks of the FADSDIET2 study was investigated. The results demonstrated that there were no differences in mitochondrial respiration parameters, ECAR, or OCR/ECAR ratio in hADSCs between the genotypes of *FADS1*-rs174550 at the 0 weeks of the study (Fig. 1A, B). Maximal respiration and ATP production were higher in differentiated hADSCs from subjects with the TT genotype when compared with the CC genotype (Fig. 1C). ECAR did not differ between the genotypes, but OCR/ECAR ratio was higher in subjects with the TT genotype when compared with the CC genotype (Fig. 1D). Additionally, bioenergetic phenotype profiling demonstrated aerobic pathway for energy production in both cell types and genotypes (Fig. 1E). Supporting the mitochondrial respiration results, mtDNA amount was higher in differentiated hADSCs in subjects with the TT genotype when compared with the CC genotype, while no differences in mtDNA amount was found in hADSCs between the genotypes of *FADS1*-rs174550 (Fig. 1F). These suggest that subjects with the higher desaturase activity of *FADS1*-rs174550 variant (the TT genotype) have enhanced mitochondrial respiration along with increased aerobic energy production in differentiated hADSCs likely through changes in mtDNA amount.

### *FADS1*-rs174550 variant is associated with modification in the expression of genes related to FA synthesis and oxidation and mitochondrial function

**Figure 2:**
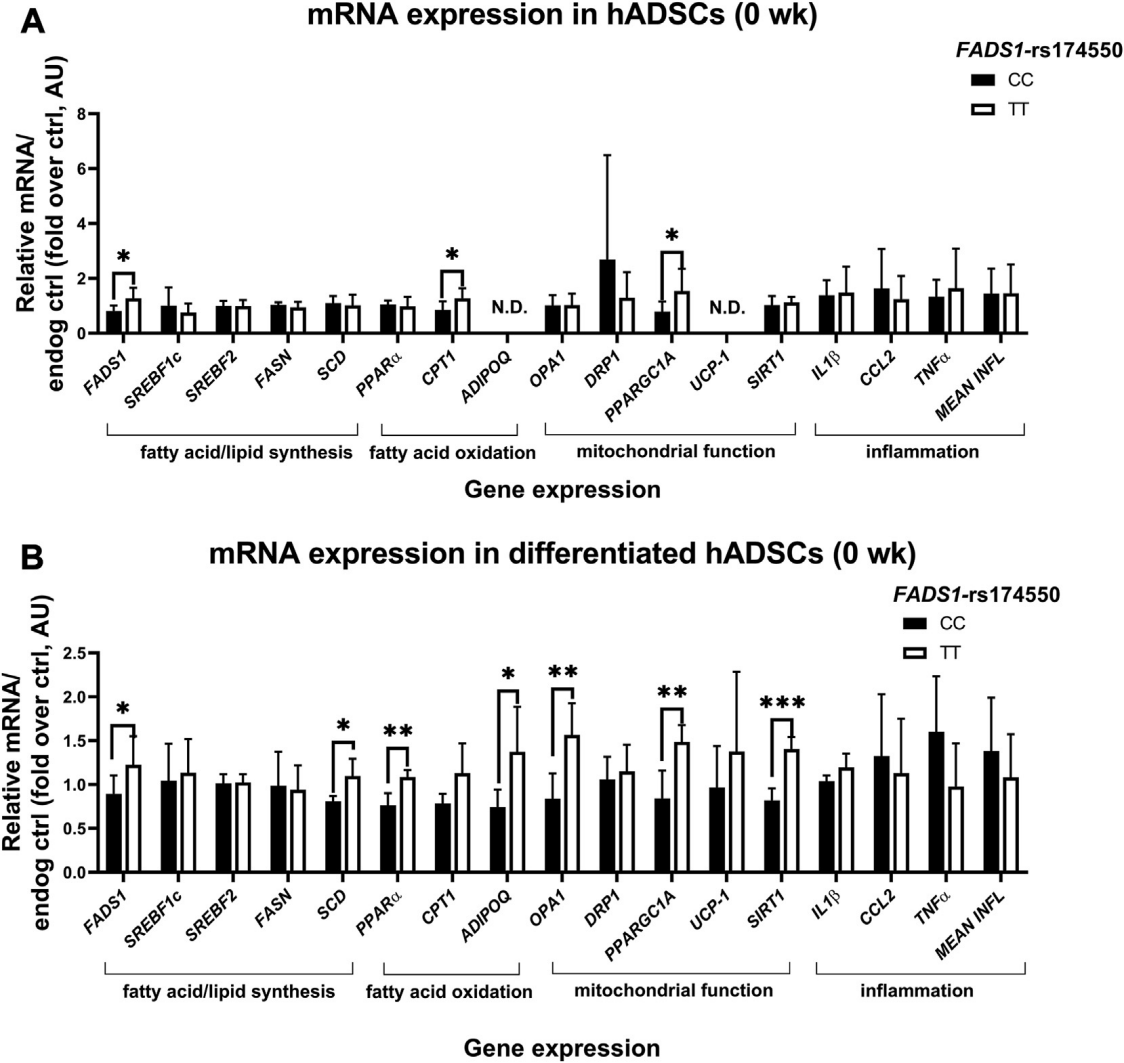
The effect of *FADS1*-rs174550 variant on the expression of genes related to fatty acid/lipid synthesis, fatty acid oxidation, mitochondrial function, and inflammation in (A) hADSCs and (B) differentiated hADSCs at 0 weeks of the FADSDIET2 study. Values are presented as mean ± SD, n = 8 independent biological replicates/genotype from male subjects, including 3 technical replicates/biological replicate. Statistics: independent samples *t* test for differences between genotypes (∗*P* < 0.05, ∗∗*P* < 0.01, ∗∗∗*P* < 0.001). FADS1, fatty acid desaturase 1; SREBF, sterol regulatory element-binding transcription factor; FASN, fatty acid synthase; SCD, stearoyl-CoA desaturase; PPARα, peroxisome proliferator-activated receptor alpha; CPT1, carnitine palmitoyltransferase 1; ADIPOQ, adiponectin; PPARGC1A, peroxisome proliferator-activated receptor gamma coactivator 1-alpha; UCP-1, uncoupling protein-1; SIRT1, sirtuin 1; IL1β, interleukin 1 beta; CCL2, C-C motif chemokine ligand 2; TNFα, tumor necrosis factor alpha; INFL, inflammation; hADSCs, human adipose-derived stromal cells; N.D., not detected.

The *FADS1*-rs174550 variant modifies the estimated enzyme activity of D5D, encoded by the *FADS1* gene and thus, we investigated whether the *FADS1* mRNA expression was modified by the *FADS1*-rs174550 variant in hADSCs and differentiated hADSCs at 0 weeks of the FADSDIET2 study. The results showed that the *FADS1* mRNA expression in hADSCs and differentiated hADSCs was higher in subjects with the TT genotype compared with the CC genotype at 0 weeks of the study (Fig. 2A and B). Additionally, the mRNA expression of carnitine palmitoyltransferase 1 (*CPT1*) and peroxisome proliferator-activated receptor gamma coactivator 1-alpha (*PPARGC1A*) was higher in subjects with the TT genotype compared with the CC genotype at 0 weeks of the study (Fig. 2A). In differentiated hADSCs, the *FADS1*-rs174550 variant was associated with modification in the expression of genes related to FA synthesis and oxidation as well as mitochondrial function, demonstrating a higher expression of stearoyl-CoA desaturase (*SCD*), peroxisome proliferator-activated receptor alpha (*PPARα*), adiponectin (*ADIPOQ*), *PPARGC1A*, optic atrophy (*OPA*)1, and sirtuin (*SIRT*)*1* in subjects with the TT genotype compared with the CC genotype at 0 weeks of the study (Fig. 2B). These support the results of mitochondrial respiration and demonstrate higher FA oxidation and mitochondrial function in subjects with the TT genotype compared with the CC genotype of *FADS1*-rs174550 also at gene expression level.

Next, we aimed to investigate whether the relationship between *FADS1*-rs174550 and FA oxidation could be reflected in clinical data. RQ value, which is used as a measure of FA oxidation (27, 28), was calculated from the gas exchange measurements through the breath by indirect calorimetry in study subjects. The results showed that there was a negative correlation between the RQ value and mtDNA amount in differentiated hADSCs from subjects with the TT (r = −0.887, *P* = 0.045), but not the CC (r = −0.629, *P* = 0.255), genotype at 0 weeks of the FADSDIET2 (Supplemental Fig. S2). This suggests a higher FA oxidation along with increased mtDNA amount in subjects with the TT genotype of *FADS1*-rs174550, complementing gene expression data.

### The *FADS1*-rs174550 variant is associated with modification in ALA-induced changes in mitochondrial respiration and mtDNA amount

**Figure 3:**
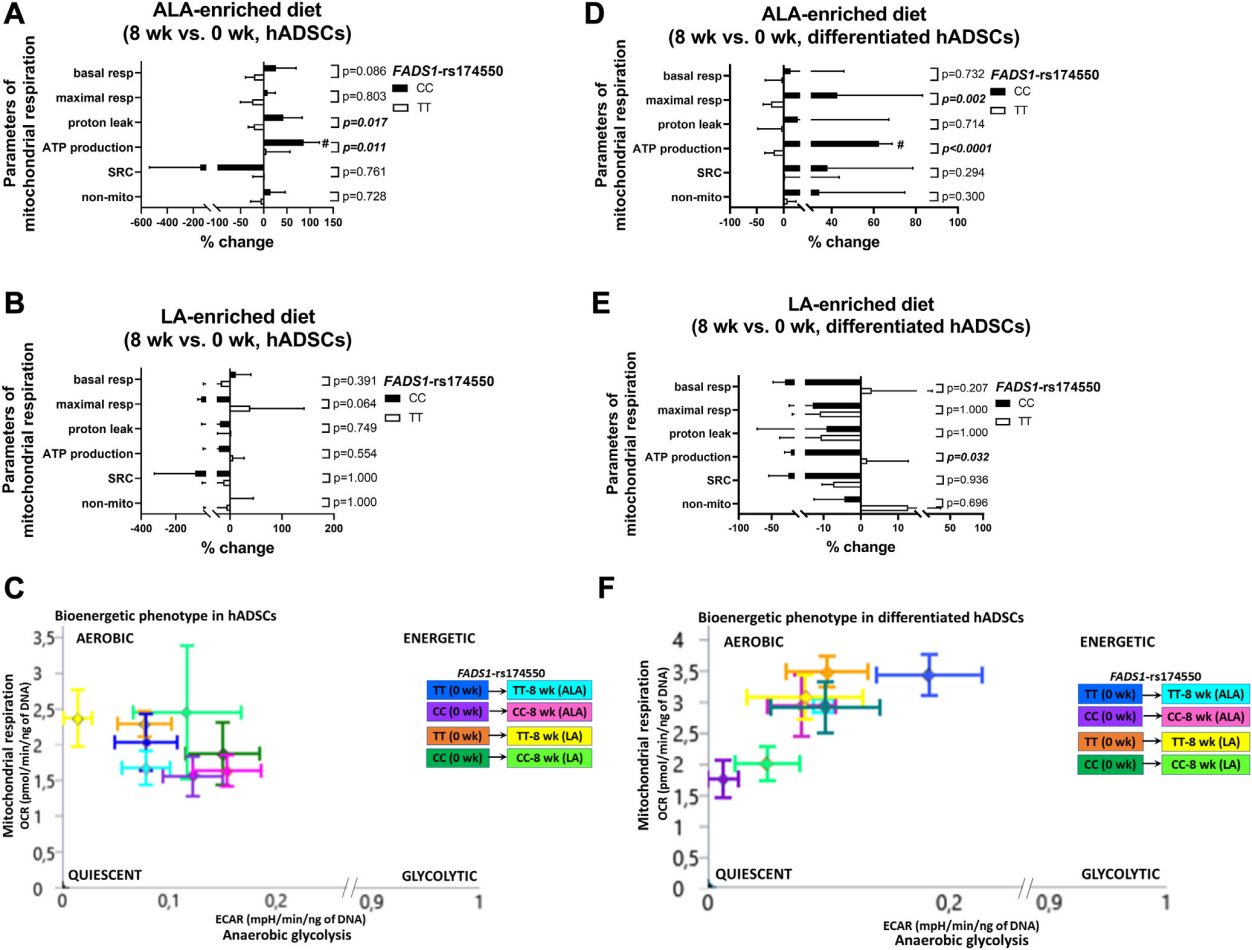
The effect of ALA- and LA-enriched diets on the parameters of mitochondrial respiration (presented as the parameters of mitochondrial respiration: basal respiration, maximal respiration, proton leak, ATP production, spare respiratory capacity (SRC), non-mitochondrial (non-mito) respiration, and the schematic representation in cellular bioenergetics phenotype profile in A-C) hADSCs and D-F) differentiated hADSCs in male subjects homozygous for the *FADS1*-rs174550. Arrows represent the direction of changes from 0 weeks (CC/TT) to the end of the intervention (CC/TT-8 weeks for ALA- and LA-enriched diets). A-B, D-E: Values are mean ± SD (n = 4/group) of percentage changes (8 weeks–0 weeks). Genotype × diet interaction (*P*-value) was tested using general linear model two-way ANOVA with Bonferroni’s multiple comparison test and within-genotype comparisons (0 weeks compared with 8 weeks) was tested with paired *t* test (#). Significant *P*-values are bolded with italics (genotype × diet interaction), #*P* < 0.05 (within-genotype). FADS1, fatty acid desaturase 1; ALA, alpha-linolenic acid; LA, linoleic acid; resp, respiration; SRC, spare respiratory capacity; non-mito, non-mitochondrial respiration; hADSCs, human adipose-derived stromal cells.

To explore whether the *FADS1*-rs174550 variant is associated with diet-induced responses to mitochondrial respiration, the effect of the ALA- and LA-enriched diets on mitochondrial respiration parameters in hADSCs and differentiated hADSCs between subjects homozygous for the *FADS1*-rs174550 variant were explored. In hADSCs, there were gene–diet interactions in proton leak and ATP production, demonstrating increased ATP production in subjects with the CC genotype in the ALA-enriched diet group (Fig. 3A). However, the LA-enriched diet did not affect the parameters of mitochondrial respiration in hADSCs (Fig. 3B). There were no changes in ECAR, OCR/ECAR ratio, or bioenergetic profile in either of the diet groups in hADSCs (Supplemental Figs. S3A, B and 3C).

In differentiated hADSCs, there were gene–diet interactions in maximal respiration and ATP production in the ALA-enriched diet group (Fig. 3D). Additionally, in line with hADSCs, ATP production was increased in subjects with the CC genotype in the ALA-enriched diet group (Fig. 3D), demonstrated also by the bioenergetic phenotype profile with a higher aerobic pathway for energy production (Fig. 3F). There was also a gene – diet interaction in ATP production in the LA-enriched diet group (Fig. 3E); however, demonstrating an opposite direction compared with the ALA-enriched diet group. There were no changes in ECAR (Supplemental Fig. S3C) or OCR/ECAR ratio (Supplemental Fig. S3D) in either of the diet groups.

**Figure 4:**
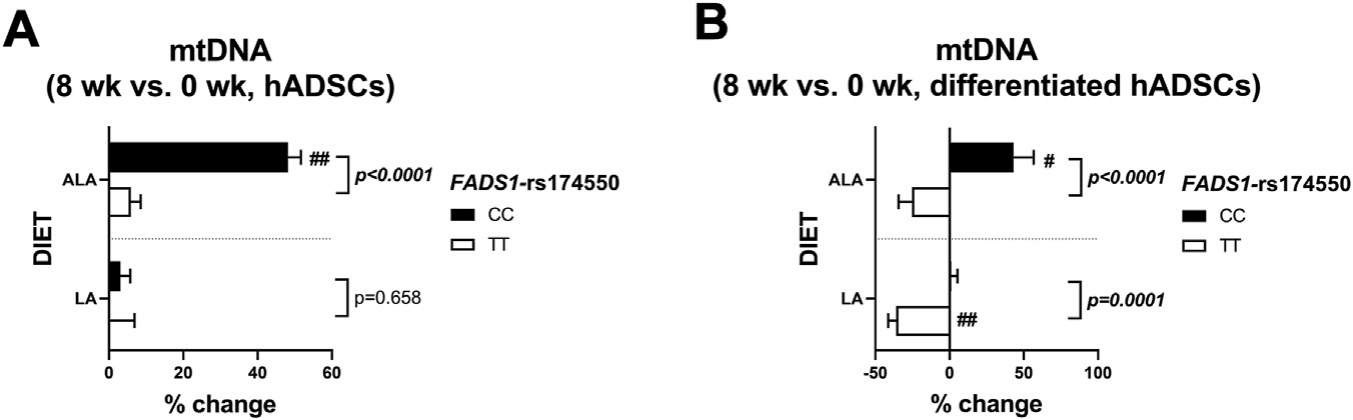
The effect of ALA- and LA-enriched diets on mitochondrial (mt)DNA amount in (A) hADSCs and (B) differentiated hADSCs in male subjects homozygous for the *FADS1*-rs174550. Values are mean ± SD (n = 4/group) of percentage changes (8 weeks–0 weeks). Genotype × diet interaction (*P*-value) was tested using general linear model two-way ANOVA with Bonferroni’s multiple comparison test and within-genotype comparisons (0 weeks compared with 8 weeks) was tested with paired *t* test (#). Significant *P*-values are bolded with italics (genotype × diet interaction), #*P* < 0.05, ##*P* < 0.01 (within-genotype). FADS1, fatty acid desaturase 1; ALA, alpha-linolenic acid; LA, linoleic acid; hADSCs, human adipose-derived stromal cells.

To investigate whether changes in mitochondrial respiration could also be reflected in mtDNA amount, the effect of the ALA- and LA-enriched diets on mtDNA amount in hADSCs and differentiated hADSCs in subjects homozygous for the *FADS1*-rs174550 were explored. As shown in Figure 4A, B, there were gene–diet interactions in mtDNA amount in the ALA-enriched diet group in both hADSCs and differentiated hADSCs, demonstrating increased mtDNA amount in subjects with the CC genotype in the ALA-enriched diet group. Additionally, there was a gene–diet interaction in mtDNA amount in differentiated hADSCs in the LA-enriched diet group (Fig. 4B), demonstrating reduced mtDNA amount in subjects with the TT genotype in the LA-enriched diet group. This suggests that *FADS1*-rs174550 could be associated with modification in the response of the ALA- but not the LA-enriched diet to mitochondrial function by stimulating mitochondrial respiration in subjects with the CC genotype, likely through increased mtDNA amount in human primary adipocytes.

### The *FADS1*-rs174550 variant is associated with modification in dietary PUFA-induced changes in the expression of genes related to FA and mitochondrial metabolism, and inflammation

**Figure 5:**
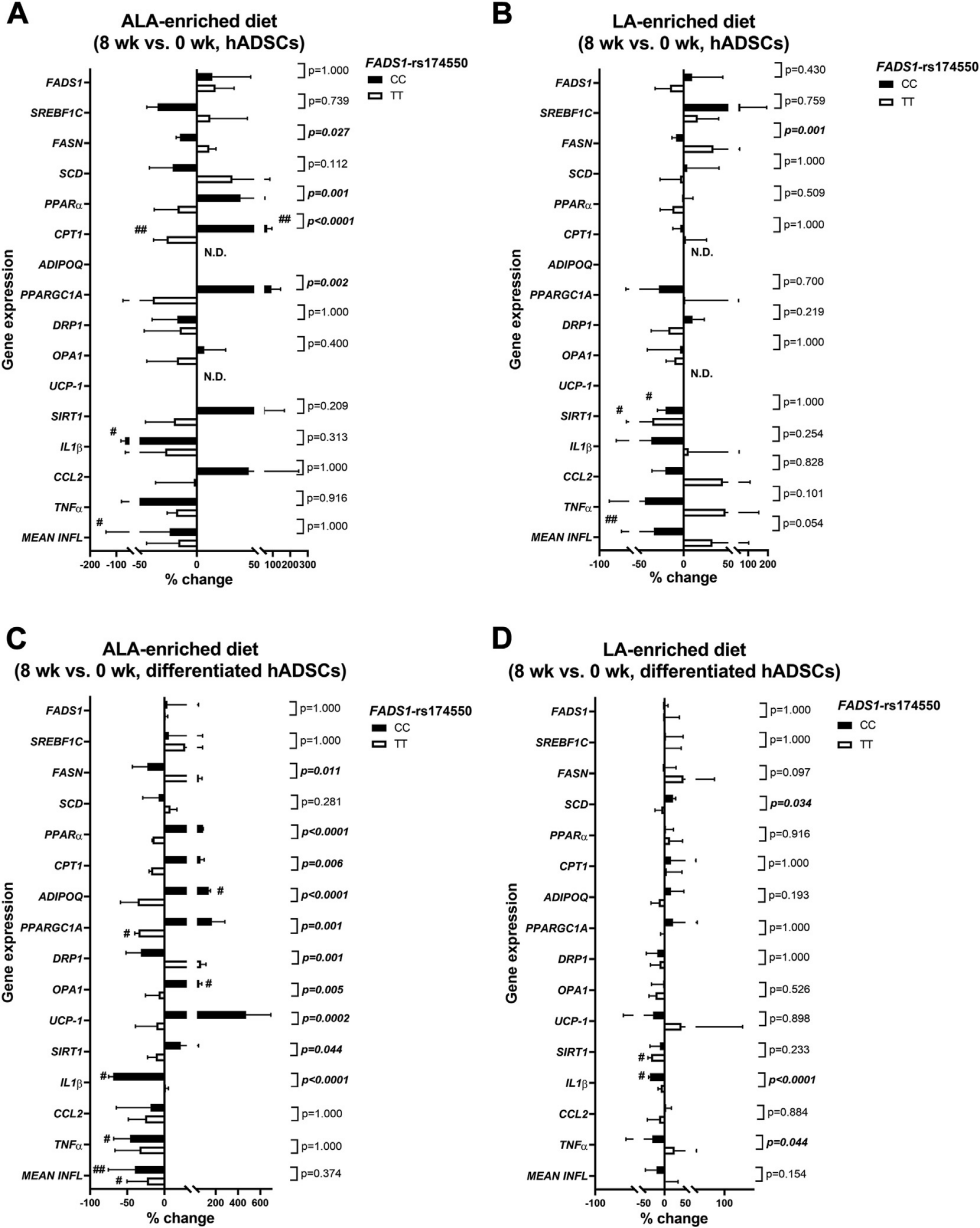
*FADS1*-rs174550 variant associates with modification in the dietary PUFA response to the expression of genes related to FA oxidation and mitochondrial function. The effect of ALA- and LA-enriched diets on gene expression levels in (A-B) hADSCs and (C-D) differentiated hADSCs, respectively, in male subjects homozygous for the *FADS1*-rs174550 variant. Values are mean ± SD (n = 4/group) of percentage changes (8 weeks–0 weeks). Genotype × diet interaction (*P*-value) was evaluated using general linear model two-way ANOVA with Bonferroni’s multiple comparison test and within-genotype comparisons (0 weeks compared with 8 weeks) was tested with paired *t* test (#). Significant *P*-values are bolded with italics (genotype × diet interaction), #*P* < 0.05, ##*P* < 0.01 (within-genotype). FADS1, fatty acid desaturase 1; PUFA, polyunsaturated fatty acid; FA, fatty acid; ALA, alpha-linolenic acid; LA, linoleic acid; SREBF, sterol regulatory element-binding transcription factor; FASN, fatty acid synthase; SCD, stearoyl-CoA desaturase; PPARα, peroxisome proliferator-activated receptor alpha; CPT1, carnitine palmitoyltransferase 1; ADIPOQ, adiponectin; PPARGC1A, peroxisome proliferator-activated receptor gamma coactivator 1-alpha; UCP-1, uncoupling protein-1; SIRT1, sirtuin 1; IL1β, interleukin 1 beta; CCL2, C-C motif chemokine ligand 2; TNFα, tumor necrosis factor alpha; INFL, inflammation; hADSCs, human adipose-derived stromal cells; N.D., not detected.

In hADCSs, there were gene–diet interactions in the expression of genes related to FA synthesis (fatty acid synthase, *FASN*) and oxidation (*PPARα*, *CPT1*), and mitochondrial biogenesis (*PPARGC1A*) in the ALA-enriched diet group (Fig. 5A). The results showed that the ALA-enriched diet increased *CPT1* mRNA expression in subjects with the CC genotype but decreased in the TT genotype. Additionally, the expression of inflammatory gene interleukin 1 beta (*IL1β*) and the mean expression of three inflammation-related genes were reduced in the ALA-enriched diet group in subjects with the CC genotype (Fig. 5A). While the LA-enriched diet induced a reduction in *SIRT1* mRNA expression in both genotypes and reduced the mean expression of inflammatory genes in subjects with the CC genotype (Fig. 5B). Further, there was a gene-diet interaction in *FASN* mRNA expression in the LA-enriched diet group (Fig. 5B).

Consistent with hADSCs, there were gene–diet interactions in the expression of genes related to FA synthesis (*FASN*) and oxidation (*PPARα*, *CPT1*), insulin sensitizer *ADIPOQ*, mitochondrial function (*PPARGC1A*, *OPA1*, dynamin-related protein 1: *DRP1*, uncoupling protein 1: *UCP-1*, *SIRT1*) and inflammation (*IL1β*) in differentiated hADSCs in the ALA-enriched diet group (Fig. 5C). The results also showed that the ALA-enriched diet increased *ADIPOQ* and *OPA1* mRNA expression in subjects with the CC genotype and decreased *PPARGC1A* mRNA expression in subjects with the TT genotype. Further, the expression of inflammatory genes, *IL1β* and tumor necrosis factor alpha (*TNFα*), were decreased in subjects with the CC genotype and the mean of inflammatory genes in both genotypes of *FADS1*-rs174550 in the ALA-enriched diet group (Fig. 5C). There were also gene-diet interactions in the expression of *SCD* and inflammatory genes *IL1β* and *TNFα* in the LA-enriched diet group (Fig. 5D). The results showed that *SIRT1* mRNA expression levels were reduced in subjects with the TT genotype while *IL1β* mRNA expression was reduced in subjects with the CC genotype in the LA-enriched diet group (Fig. 5D). Altogether, these suggest that the ALA-enriched diet compared with LA-enriched diet is more beneficial in subjects with the CC, but not in the TT genotype, demonstrating improved FA oxidation and mitochondrial metabolism and at the same time attenuated inflammation at the gene expression level.

### *FADS1*-rs174550 associates with modification in the correlation between plasma FA proportions and mtDNA amount in scAT of subjects with obesity

Next, to validate the relationship between ALA and LA with mDNA amount, we explored whether *FADS1*-rs174550 associates with the relationship between the dietary intake of ALA and LA with mtDNA amount in scAT in subjects with obesity of the KOBS study. The results showed that there was a positive correlation (r = 0.820, *P* = 0.046) between the proportion of ALA in plasma PL fraction and mtDNA amount in scAT (adjusted to age and BMI) in subjects with the CC genotype of *FADS1*-rs174550 (Supplemental Fig. S4A). In participants with the TT genotype, the proportion of ALA in plasma PL fraction did not correlate (r = 0.276, *P* = 0.385) with mtDNA in scAT (adjusted to age and BMI, Supplemental Fig. S4A). Furthermore, there were no correlations between the proportion of LA in plasma PL fraction and mtDNA amount in scAT in either of the genotypes (Supplemental Fig. S4B). Collectively, these results support the finding that the *FADS1* variant could be associated with modification in the interaction between dietary n-3 PUFAs and mtDNA amount in scAT.

## Discussion

Our results demonstrate that the *FADS1*-rs174550 could be associated with modification in mitochondrial bioenergetics in differentiated hADSCs. Additionally, the *FADS1*-rs174550 could be associated with modification in the response of a dietary n-3 ALA-enriched diet to mitochondrial metabolism and inflammation. The results demonstrated improved mitochondrial respiration, mtDNA amount, and the expression of genes related to FA oxidation and mitochondrial function, while the expression of genes related to inflammation was reduced in participants with the CC genotype in the ALA-enriched diet group. While the LA-enriched diet had only a few or even opposite effects on mitochondrial metabolism when compared with the ALA-enriched diet group. Therefore, the ability of dietary ALA to stimulate mitochondrial respiration in white adipose tissue may demonstrate a personalized therapeutic potential to tackle obesity through improved white adipose tissue bioenergetics.

Previous studies have shown that beyond its role in PUFA biosynthesis, *FADS1* could also be active in mitochondria (15, 33, 34). Here, we demonstrate for the first time that the *FADS1*-rs174550 variant could be associated with modification in mitochondrial function in hADSCs. The results demonstrated that mitochondrial respiration in differentiated hADSCs was higher in subjects with the TT genotype compared with the CC genotype. This could be explained by changes in mitochondrial dynamics, as mtDNA amount and the expression of *PPARGC1A* and *OPA1*, key regulators of mitochondrial biogenesis (35) and fusion (36), respectively, were higher in subjects with the TT compared with the CC genotype, suggesting differences in mitochondrial dynamics and efficiency between the genotypes of *FADS1*-rs174550. Additionally, the mRNA expression data demonstrated higher expression of genes related to FA oxidation (*PPARα*), insulin sensitizer (*ADIPOQ*), and mitochondrial function (*SIRT1*), mainly in differentiated hADSCs. Our results showed an inverse correlation between the RQ value and mtDNA amount in differentiated hADSCs in subjects with the TT genotype. As low RQ values reflect high FA oxidation, this could link a higher mtDNA amount with improved FA oxidation in subjects with the TT genotype, complementing the mRNA results of genes related to FA oxidation. Interestingly, genetic variation in the *FADS2* gene (rs174576) has also been shown to be associated with whole-body fat oxidation (37), indicating that genetic variations in the *FADS* genes may play a role in energy metabolism. Overall, our results suggest that the *FADS1*-rs174550 could be associated with modification in energy metabolism in white adipose tissue as mitochondrial respiration and dynamics in combination with FA oxidation in differentiated hADSCs differed between the genotypes of *FADS1*. However, the mechanism is yet unknown, but one explanation could be a higher synthesis of n-3 PUFA EPA, with beneficial effects on mitochondrial function, in subjects with TT genotype and higher D5D activity. Additionally, D5D/FADS1 has been shown to be involved in cytosolic NAD/NADH recycling (14) which in mitochondria controls mitochondrial respiration (38). However, these need to be clarified by further investigations. Previous studies have reported that genetic variations in the *FADS1* gene have no impact on serum hs-CRP levels (21) or adipose tissue inflammatory status in obesity (39, 40). We also demonstrated that the *FADS1*-rs174550 did not modify the expression of genes related to inflammation, suggesting that the *FADS1* has no modulatory effect on inflammation in adipocytes from healthy individuals.

Next, we illustrated that the responses of dietary PUFAs to mitochondrial function differed between the genotypes of *FADS1*-rs174550. The results showed several gene–diet interactions in the parameters of mitochondrial respiration, including proton leak and ATP production in hADSCs and maximal respiration and ATP production in differentiated hADSCs in response to ALA-enriched diet. Additionally, ALA-, but not LA-, enriched diet increased ATP production in both cell types only in subjects with the CC genotype of *FADS1*-rs174550. PUFAs serve as substrates for mitochondrial respiration (41, 42). However, little is known about ALA-induced responses to mitochondrial function. You *et al.* (43) have demonstrated that an ALA-enriched butter diet promotes the activation of thermogenesis and mitochondrial biogenesis in the brown adipose tissue of mice with obesity. Although LA is an important component of mitochondrial structure (42), its effects on mitochondrial function are controversial. A study by Astorg *et al.* (44) showed that LA had little or no effect on mitochondrial respiratory parameters in rats fed with or without LA. On the other hand, dietary intake of LA has been shown to improve mitochondrial respiratory mechanisms in mouse models with heart failure (45). Thus, our results provide new evidence that the *FADS1* variant associates with modification in ALA, but not LA, -induced effects on mitochondrial respiration in human adipocytes.

Notably, the observed beneficial changes in mitochondrial respiration in the ALA-enriched diet group were, at least partially, a result of the induction of mitochondrial biogenesis, dynamics, and number. Since there were gene-diet interactions in the expression of genes related to mitochondrial biogenesis and fusion (*PPARGC1A*, *SIRT1*, *OPA1*, and *UCP-1*) towards increased expression in subjects with the CC genotype. Additionally, there were gene–diet interactions in mtDNA amount in both cell types (hADSC and differentiated hADSC), demonstrating increased mtDNA amount in subjects with the CC genotype in the ALA-enriched diet group. Whereas mtDNA amount was reduced in subjects with the TT genotype in the LA-enriched diet group. Interestingly, mitochondrial fusion mediator OPA1 has been shown to stimulate mitochondrial respiration through the regulation of mtDNA amount (36). Therefore, it could be possible that an ALA-enriched diet improves mitochondrial respiration through increased mitochondrial fusion and subsequent effects on biogenesis/mtDNA amount in subjects with the CC genotype.

We replicated the relationship between ALA and mtDNA in a validation cohort of subjects with obesity participating in the KOBS study. Based on the results, there was a positive correlation between the proportion of ALA in plasma PL fraction and mtDNA amount in scAT in subjects with the CC genotype of *FADS1*-rs174550, supporting the results in the FADSDIET2 study. This suggests that ALA-induced effects on mitochondrial respiration could be mediated through increased mitochondrial biogenesis.

Dietary FAs are able to modify mitochondrial function through metabolic gene regulation by increasing the expression of genes related to FA oxidation (42). Consistent with this, our results showed gene–diet interactions in the expression of genes related to FA oxidation (*PPARα* and *CPT1*) and insulin sensitizer (*ADIPOQ*) in response to the ALA-enriched diet. ALA is shown to be a substrate for FA oxidation and energy production (46) and an LA-enriched diet has been shown to reduce the expression of genes involved in FA oxidation and increase the expression of genes related to lipogenesis in high-fat-fed mice (47). *PPARα* is a master regulator of FA oxidation (42) and CPT1, which is regulated by the *PPARα,* is an important factor in catalyzing the rate-limiting step in mitochondrial FA beta-oxidation (48). ADIPOQ, in addition to its role as an insulin sensitizer, increases the transport of FAs into mitochondria by blocking the inhibition of CPT1 and thus promoting FA oxidation (49). Our results did not show significant changes in mitochondrial respiration or the expression of genes related to mitochondrial function and FA oxidation in response to the LA-enriched diet. There is no clear explanation for this, but it is possible that this phenomenon is because LA could be synthesized into AA and subsequent AA-derived lipid mediators, which have been shown to inhibit mitochondrial function (50) and adipocyte browning (51) and induce de novo FA synthesis in the liver (47). Furthermore, LA- and AA-derived metabolites are shown to be associated with enhanced oxidative stress, macrophage activation, and inflammation (10), suggesting defective effects on mitochondrial function.

Our results demonstrated that the *FADS1*-rs174550 associated with modification in dietary PUFA-induced effects on the expression of genes related to inflammation in hADSCs. The expression of genes related to inflammation was reduced in both diet groups in subjects with the CC but not the TT genotype of *FADS1*-rs174550. However, the response of an ALA-enriched diet to inflammation was stronger in subjects with the CC genotype and specifically in differentiated hADSCs. PUFAs, especially n-3 PUFAs, are shown to have beneficial effects on inflammation. ALA-enriched butter diet has been shown to reduce high-fat diet-induced adipose tissue inflammation in mice (8). Additionally, ALA has been shown to decrease hs-CRP, a marker of systemic inflammation in humans (52, 53). Thus, the *FADS1*-rs174550 could be associated with modification in PUFA-induced responses to inflammation, showing beneficial effects with an ALA-enriched diet in subjects with the CC genotype.

Our unique study design combines data from individuals with normal weight (FADSDIET2) and obesity (KOBS), which were chosen based on their specific *FADS1* variant. However, future studies should address the limitations of the current study design. We acknowledge that the sample size is small and that the results consisted only of middle-aged men. Thus, these results cannot be generalized to women or a younger population. Additionally, statistical power due to the small sample size may bias our indirect calorimetry correlation results, and thus, those need to be interpreted with caution. While hADSCs were collected from the same scAT depot, the reality is that the hADSCs may not be identical due to variability in stromal cells from the site of collection and culturing conditions, etc. Thus, we cannot rule out the possibility that some of the differences observed between weeks 0 and 8 may simply be due to differences in cell populations.

Overall, at baseline, the TT genotype, with increased D5D activity, had higher mitochondrial activity compared with the CC genotype. However, the effect of an ALA-enriched diet switched this. This could be because even 30%–60% of ingested ALA may be targeted to energy production (54) and thus, the greater ALA availability coupled with reduced D5D activity in the CC genotype in the ALA-enriched diet group promotes FA oxidation and mitochondrial activity. In conclusion, the current data provide novel evidence for the first time that the *FADS1*-rs174550 could be associated with modification in mitochondrial function in hADSCs. Additionally, this study suggests that the *FADS1*-rs174550 could be a genetic marker to identify subjects who are most suitable to receive a dietary n-3 PUFA supplementation, establishing a novel personalized therapeutic strategy to improve mitochondrial function and thus tackle the development of metabolic diseases.

## Data availability

The data that support the findings of this study are not openly available due to reasons of sensitivity.

## Supplemental data

This article contains supplemental data.

## Conflicts of interest

The authors declare that they have no conflicts of interest regarding the contents of this article.
