# Supplementary material for: Modification in mitochondrial function is associated with the FADS1 variant and its interaction with alpha-linolenic acid-enriched diet—An exploratory study
Source: J Lipid Res. 2024 Aug 31;65(10):100638. doi: 10.1016/j.jlr.2024.100638 (PMC11459653; doi:10.1016/j.jlr.2024.100638)
Supplement: Supplemental Figures S1-S4 and Tables — S1, S2 [file mmc1.docx]

**Modification in mitochondrial function is associated with the *FADS1* variant and its interaction with alpha-linolenic acid-enriched diet - an exploratory study**

**Supplementary Table S1**. Main characteristics of the male study participants, from which the data of plasma fatty acid proportions in phospholipid fraction and mitochondrial DNA in subcutaneous adipose tissue were available, between subjects homozygous (CC, n=28; TT, n=39) for the genotypes of *FADS1*-rs174550 variant at the baseline of the Kuopio Obesity Surgery study

|  | TT (n=39), major allele | CC (n=28), minor allele | p value^a^ |
| --- | --- | --- | --- |
| Gender (males/females) | 14/25 | 8/20 | 0.739 |
| Age (years) | 47.4±9.1 | 47.4±9.0 | 1.000 |
| Body Weight (kg) | 128.4±17.9 | 125.3±18.1 | 1.000 |
| BMI (kg m-2) | 45.0±6.1 | 43.4±5.6 | 0.900 |
| Waist circumference (cm)^b^ | 129.2±11.7 | 135.7±19.4 | 1.000 |
| fP glucose (mmol L-1) | 6.7±1.9 | 6.2±1.2 | 1.000 |
| fP insulin (mU L-1) | 20.0±12.9 | 17.5±9.0 | 1.000 |
| fS total cholesterol (mmol L-1) | 4.4±1.1 | 4.5±1.0 | 1.000 |
| fS LDL cholesterol (mmol L-1) | 2.6±0.9 | 2.7±1.0 | 1.000 |
| fS HDL cholesterol (mmol L-1) | 1.1±0.3 | 1.1±0.4 | 1.000 |
| fS triglycerides (mmol L-1) | 1.6±0.6 | 1.6±0.7 | 1.000 |
| Use of statins, n (%) | n/a | n/a | n/a |
| Use of anti-inflammatory drugs, n (%) | n/a | n/a | n/a |
| Use of beta-blockers, n (%) | n/a | n/a | n/a |
| Mean ± SD; fP, fasting plasma; fS, fasting serum; FADS1, fatty acid desaturase; a) One-Way ANOVA for continuous variables or 𝜒2 test for categorical variables; b) TT: n=7, CC: n=6; n/a, non-available | | | |

**Supplementary Table S2.** Details of primers used in quantitative real-time PCR for mRNA expression levels and for the detection of mitochondrial (mt) and nuclear (nc) DNA.

| **Gene symbol** | **Gene name** | **Sequence details** | **Chemistry** | **Target** | |
| --- | --- | --- | --- | --- | --- |
| *PPIA* | cyclophilin A1 | Hs99999904_m1 | TaqMan | mRNA |  |
| *CPT1* | carnitine palmitoyltransferase 1 | Hs00189258_m1 | TaqMan | mRNA |  |
| *FADS1* | fatty acid desaturase 1 | Hs01096545_m1 | TaqMan | mRNA |  |
| *ADIPOQ* | adiponectin | Hs00605917_m1 | TaqMan | mRNA |  |
| *PPARGC1A* | peroxisome proliferator-activated receptor gamma coactivator 1-alpha | Hs01016719_m1 | TaqMan | mRNA |  |
| *CCL2* | C-C motif chemokine ligand 2 | Hs00234140_m1 | TaqMan | mRNA |  |
| *UCP-1* | uncoupling protein 1 | Hs00222453_m1 | TaqMan | mRNA |  |
| *TNFα* | tumor necrosis factor alpha | Hs00174128_m1 | TaqMan | mRNA |  |
| *IL1β* | interleukin 1 beta | Hs01555410_m1 | TaqMan | mRNA |  |
| *SCD* | stearoyl-CoA desaturase | Hs01682761_m1 | TaqMan | mRNA |  |
| *PPARα* | peroxisome proliferator-activated receptor alpha | forward: TCCACGCGTGTGAAGGCTGC,  reverse: AGCTGCGGTCGCACTTGTCA | SYBR Green | mRNA |  |
| *FASN* | fatty acid synthase | forward: TTCTACGGCTCCACGCTCTTCC,  reverse: GAAGAGTCTTCGTCAGCCAGGA | SYBR Green | mRNA |  |
| *SREBF1c* | sterol regulatory element-binding transcription factor 1c | forward: CCATGGATTGCACTTTCGAA,  reverse: GGCCAGGGAAGTCACTGTCTT | SYBR Green | mRNA |  |
| *SREBF2* | sterol regulatory element-binding transcription factor 2 | forward: TGGCACGCTGCAGACCCTTG,  reverse: TGAGGCTGGACCAGGACCGG | SYBR Green | mRNA |  |
| *RPLPO* | ribosomal protein lateral stalk subunit P0 | forward: GGCGACCTGGAAGTCCAACT,  reverse: CCATCAGCACCACAGCCTTC | SYBR Green | mRNA |  |
| *SIRT1* | sirtuin 1 | forward: TAGACACGCTGGAACAGGTTGC, reverse: CTCCTCGTACAGCTTCACAGTC | SYBR Green | mRNA |  |
| *16S rRNA* | mitochondrially encoded 16S ribosomal RNA | forward: GGGGCGACCTCGGAGCAGAA, reverse: ATAGCGGCTGCACCATCGGGA | SYBR Green | mtDNA |  |
| *CYTB* | mitochondrially encoded cytochrome B | forward: GCCTGCCTGATCCTCCAAAT,  reverse: AAGGTAGCGGATGATTCAGCC | SYBR Green | mtDNA |  |
| *D-loop* | mitochondrially encoded D-loop | forward: CATCTGGTTCCTACTTCAGGG, reverse: CCGTGAGTGGTTAATAGGGTG | SYBR Green | mtDNA |  |
| *HBB* | Hemoglobin Subunit Beta | forward: CAGGTACGGCTGTCATCAGTTAG, reverse: CATGGTGTCTGTTTGAGGTTGCT | SYBR Green | ncDNA |  |
| *APP* | Amyloid Beta Precursor Protein | forward: TGTGTGCTCTCCCAGGTCTA,  reverse: CAGTTCTGGATGGTCACTGG | SYBR Green | ncDNA |  |
| *B2M* | Beta-2-Microglobulin | forward: TGCTGTCTCCATGTTTGATGTATCT, reverse: TCTCTGCTCCCCACCTCTAAGT | SYBR Green | ncDNA |  |


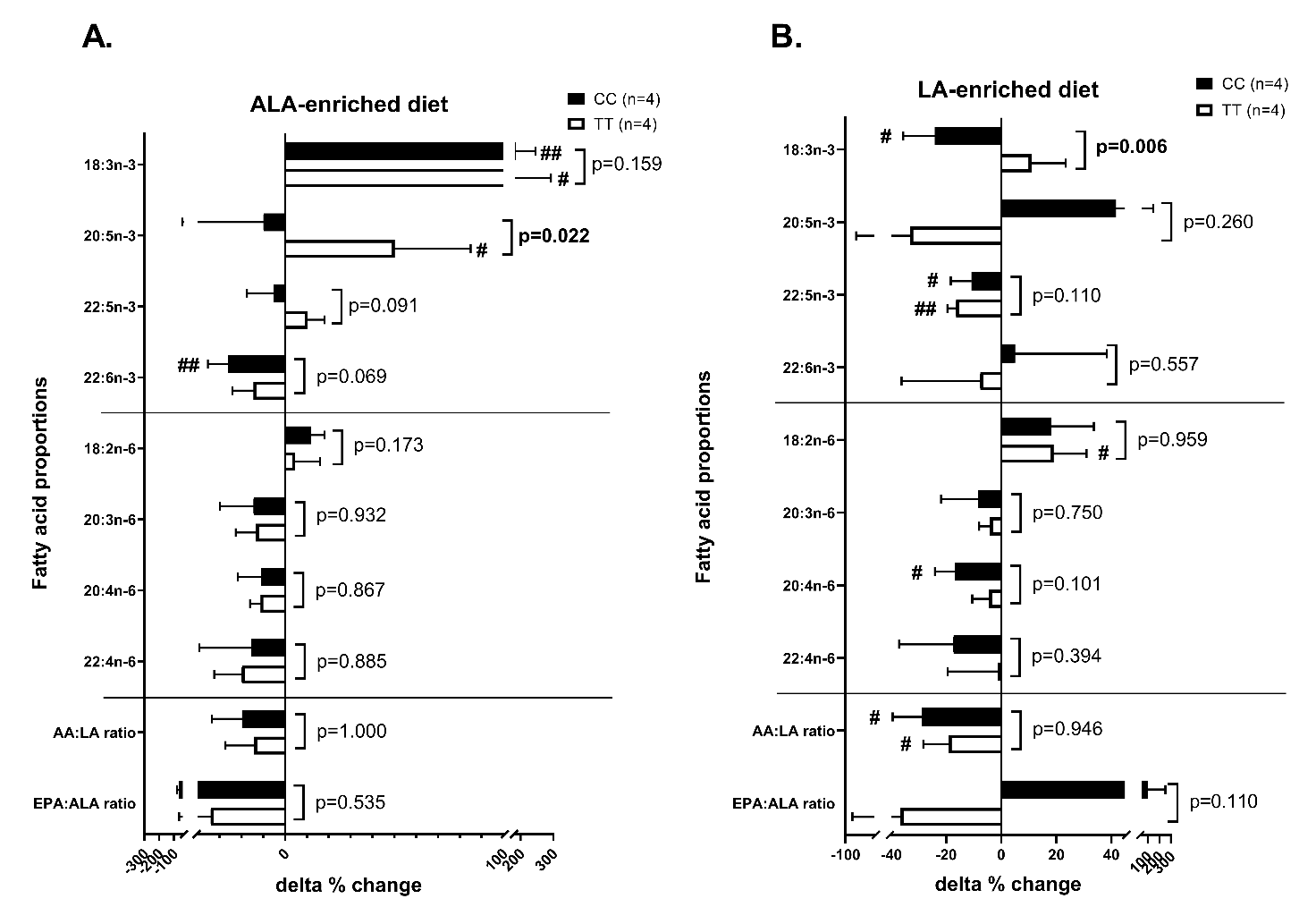


**Supplementary Figure S1.** Changes in proportions of n-6 and n-3 polyunsaturated fatty acids in plasma phospholipid fraction in male subjects homozygous for the *FADS1*-rs174550 genotypes and intervention diets. Values are mean±SD (n=4/group) of percentage changes (8 week–0 week). Genotype x diet interaction (p-value) was tested using a repeated-measures general linear model with Bonferroni’s multiple comparison test and within-genotype comparisons (0 wk compared with 8 wk) were tested with paired t-test (#). Significant p-values are bolded (genotype x diet interaction), #p<0.05, ##p<0.01 (within-genotype). FADS1, fatty acid desaturase 1; ALA, alpha-linolenic acid; LA, linoleic acid; AA, arachidonic acid; EPA, eicosapentaenoic acid

**
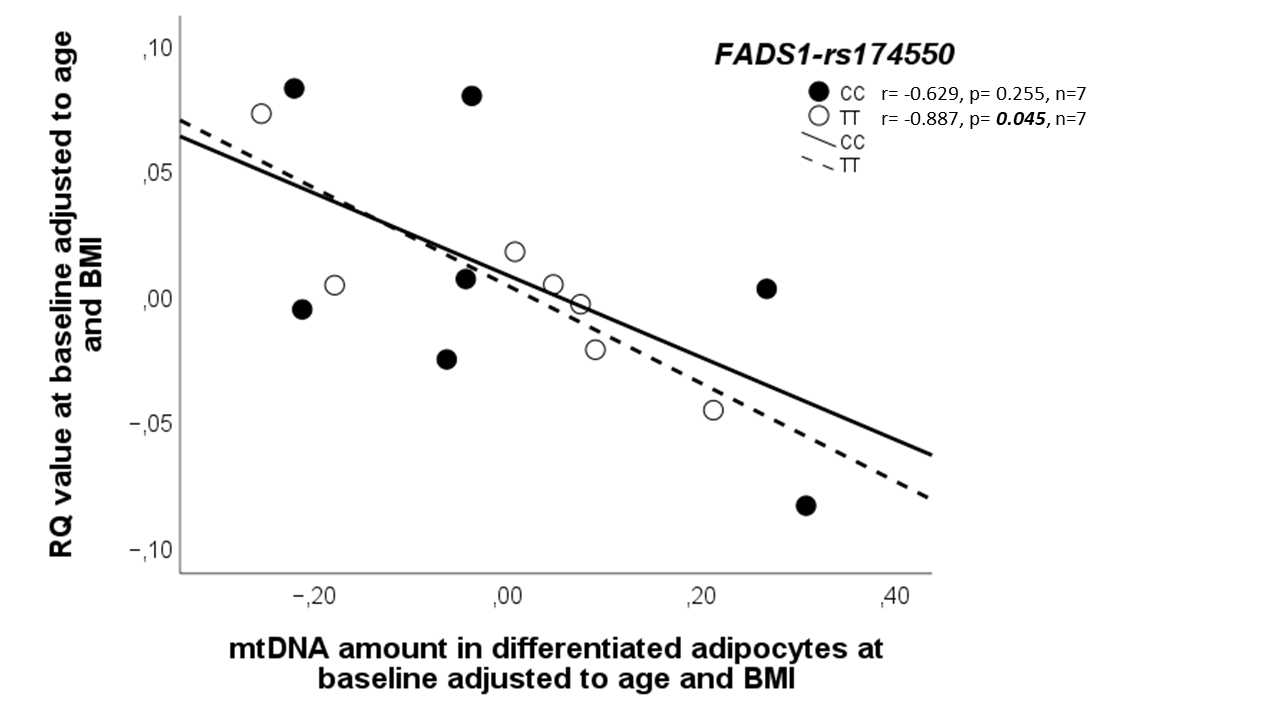
**

**Supplementary Figure S2.** Scatter plot demonstrating age and BMI adjusted Pearson’s partial correlations between respiratory quotient (RQ) value and mitochondrial (mt)DNA amount in differentiated hADSCs in male subjects with the CC (n=7) and the TT (n=7) genotype of *FADS1*-rs174550 at baseline (0 wk) of the FADSDIET2 study. Statistically significant p value: bolded with italics. BMI, body mass index; FADS1, fatty acid desaturase 1; hADSCs, human adipose-derived stromal cells

**Supplementary Figure S3.** The effect of ALA- and LA-enriched diets on the parameters of mitochondrial respiration ECAR (anaerobic glycolysis) and OCR/ECAR ratio in **A-B)** hADSCs and **C-D)** differentiated hADSCs in male subjects homozygous for the *FADS1*-rs174550. Values are mean±SD (n=4/group). Genotype x diet interaction (p-value) was tested using general linear model two-way ANOVA with Bonferroni’s multiple comparison test and within-genotype comparisons (0 wk compared with 8 wk) was tested with paired t-test (#). FADS1, fatty acid desaturase 1; ALA, alpha-linolenic acid; LA, linoleic acid; hADSCs, human adipose-derived stromal cells; OCR, oxygen consumption rate; ECAR, extracellular acidification rates

**
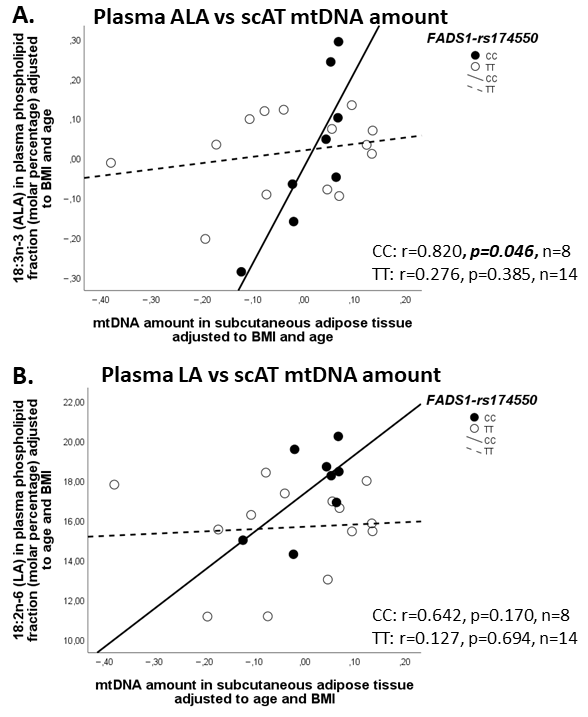
**

**Supplementary Figure S4.** Logarithmically (log) transformed scatter plots demonstrating age and BMI adjusted Pearson’s partial correlations between the proportions of **A)** plasma alpha-linolenic acid (ALA) and **B)** linoleic acid (LA) in phospholipid fraction with subcutaneous adipose tissue (scAT) mitochondrial DNA (mtDNA) amount in male subjects homozygous (CC, n=8; TT, n=14) for the *FADS1*-rs174550 variant with obesity in the Kuopio Obesity Surgery (KOBS) study. Statistically significant p value: bolded with italics. BMI, body mass index; FADS1, fatty acid desaturase 1; ALA, alpha-linolenic acid; LA, linoleic acid
